# A Wearable-Sensor System with AI Technology for Real-Time Biomechanical Feedback Training in Hammer Throw †

**DOI:** 10.3390/s23010425

**Published:** 2022-12-30

**Authors:** Ye Wang, Gongbing Shan, Hua Li, Lin Wang

**Affiliations:** 1 CAS Key Laboratory of Human-Machine Intelligence-Synergy Systems, and Guangdong-Hong Kong-Macau Joint Laboratory of Human-Machine Intelligence-Synergy Systems, Shenzhen Institutes of Advanced Technology (SIAT), Chinese Academy of Sciences (CAS), Shenzhen 518055, China; 2 Department of Mathematics & Computer Science, University of Lethbridge, Lethbridge, AB T1K3M4, Canada; 3 Department of Kinesiology & Physical Education, University of Lethbridge, Lethbridge, AB T1K3M4, Canada

**Keywords:** deep learning, hammer throw, real-time biomechanical feedback, wearable devices, wireless sensor systems

## Abstract

Developing real-time biomechanical feedback systems for in-field applications will transfer human motor skills’ learning/training from subjective (experience-based) to objective (science-based). The translation will greatly improve the efficiency of human motor skills’ learning and training. Such a translation is especially indispensable for the hammer-throw training which still relies on coaches’ experience/observation and has not seen a new world record since 1986. Therefore, we developed a wearable wireless sensor system combining with artificial intelligence for real-time biomechanical feedback training in hammer throw. A framework was devised for developing such practical wearable systems. A printed circuit board was designed to miniaturize the size of the wearable device, where an Arduino microcontroller, an XBee wireless communication module, an embedded load cell and two micro inertial measurement units (IMUs) could be inserted/connected onto the board. The load cell was for measuring the wire tension, while the two IMUs were for determining the vertical displacements of the wrists and the hip. After calibration, the device returned a mean relative error of 0.87% for the load cell and the accuracy of 6% for the IMUs. Further, two deep neural network models were built to estimate selected joint angles of upper and lower limbs related to limb coordination based on the IMUs’ measurements. The estimation errors for both models were within an acceptable range, i.e., approximately ±12° and ±4°, respectively, demonstrating strong correlation existed between the limb coordination and the IMUs’ measurements. The results of the current study suggest a remarkable novelty: the difficulty-to-measure human motor skills, especially in those sports involving high speed and complex motor skills, can be tracked by wearable sensors with neglect movement constraints to the athletes. Therefore, the application of artificial intelligence in a wearable system has shown great potential of establishing real-time biomechanical feedback training in various sports. To our best knowledge, this is the first practical research of combing wearables and machine learning to provide biomechanical feedback in hammer throw. Hopefully, more wearable biomechanical feedback systems integrating artificial intelligence would be developed in the future.

## 1. Introduction

In most sports, limb coordination is an essential factor influencing the performance of a motor skill, but it is very difficult to be quantified [1,2,3]. Obtaining reliable kinematic and kinetic parameters, such as joint angles, applied forces, etc., and further performing 3D motion analysis can help to reveal the related hidden information. Such kind of biomechanical feedback training would be quite useful to facilitate a motor skill’s learning procedure and improve its training efficiency. Currently, it has been agreed on that optoelectronic motion capture systems can provide quite reliable kinematic data [4,5,6,7]. However, there are several drawbacks of the optoelectronic method, for instance, lab-based, high cost, complicated operation, time-consuming course on data collection and processing, movement constraints induced by dozens of motion-capture markers. These drawbacks have limited their practicality and application in field [8,9], especially in those sports involving high speed and complex motor skills, e.g., men’s hammer throw, which is very difficult to calibrate and set up an optoelectronic motion capture system outside a lab. Hence, it is time to develop new motion analysis method that can overcome these difficulties for practitioners. Wearable technology has a great potential for the development.

Men’s hammer throw, like men’s 100 m sprinting, has a long history. Yet, unlike men’s 100 m sprinting and other events of field and track, its world record has ceased to refresh since 1986. One major reason is that hammer-throw coaches and athletes still get used to a traditional training style—experience-based approach. The coaches’ verbal instructions are usually based on their own subjective experience and observation [10]. Therefore, an evidence-based training has a considerable competency to help the hammer-throw coaches to establish a more efficient training method. One option for developing the evidence-based training is to apply innovative technologies, such as wearable systems and machine learning in coaching practice. Such an approach would help the elite athletes to activate their potential capabilities and stretch human biological boundaries [11,12,13].

Among the wearable sensor approaches, a portable motion capture system with multiple inertial measurement units (IMUs) is gaining more interests recently [14,15,16]. The existing inertial motion capture systems in market, such as Xsens^®^ MVN system, require at least seventeen IMU modules/trackers (i.e., each IMU module/tracker needs to be placed on head, sternum, shoulders, upper arms, fore arms, hands, pelvis, upper legs, lower legs and feet) to construct a complete human motion model for comprehensive biomechanical analysis [17,18,19]. These systems are still not practical, because the movement constraints induced by over 17 IMUs for a full-body model are reality challenges for providing coach-friendly and real-time biomechanical feedback in the hammer-throw field training. In short, the current wearable technology cannot meet the practical demands in hammer-throw training. The existing biomechanical feedback approaches/systems have their own advantages and limitations as shown in Table 1. Therefore, developing task-specific biomechanical feedback systems can usually be an ultimate solution to improve training performance [20].

The summary table has demonstrated that wearable-sensor systems have their great potential (i.e., coach-friendly/easy-to-use) of being used in daily trainings [21,22]. The widely used wearable sensors for sporting dynamic tasks include but not limited to IMUs, load cells/pressure sensors, bend/angle sensors, and electromyography (EMG) sensors [12,20,23,24]. Yet, the common challenge of these sensor technologies is how to find/reach a balance between the diversity of full-body kinematic and kinetic data and the flexibility of human body movements while using various sensors. In other words, it is ideal to use as few wearable devices as possible to acquire as many biomechanical parameters as possible. One possible way for meeting the practical requirement is to combine wearables and machine learning in establishing real-time biomechanical feedback training in the fast and dynamic elite sports [25].

### 1.1. Current Wearable Progress in Human Motor Skill Analysis

Several studies in movement biomechanics have already put great effort into reducing the sensor number and weight of wearable devices [26,27,28]. Liu et al. have designed and developed a wearable device by integrating micro tri-axis flow sensors with micro IMUs to capture dynamic motion of human limbs [5]. A neural network model has been established to reveal the motion feature of lower limbs’ coordination between thigh and shank during human walking. The study has found that just using one wearable device placed on shank is good enough to estimate the lower limbs’ kinematic parameters. Oubre et al. have prototyped a low-cost wearable system with IMUs, string-based bend/angle sensors and six force-sensitive resistors (i.e., instrumented insoles) to monitor various health conditions by combining with machine learning modeling [29]. The random forest algorithm has been used in their machine learning models for estimating ground reaction force (GRF) and center of pressure. Stetter et al. have proposed an approach to estimate biomechanical parameters on knee joint using two customized IMU devices combining an artificial neural network (ANN) model [30]. The IMU data have been used as input variables and the optical motion capture data are used as output variables in their ANN model for estimating external knee flexion and adduction moments. It is worth to mention that all the developments are limited to normal human movements, i.e., they can hardly be applied in the fast and dynamic elite sports, such as the hammer throw.

### 1.2. Hammer-Throw Related Work

Our previous work [31] has introduced a customized wearable device designed for the hammer-throw training in the field. The device was developed based on Arduino Mega board. XBee RF modules using IEEE 802.15.4 protocol were applied as the wireless communication method. An infrared sensor and a load cell were used for acquiring the vertical displacements from the waist to the ground and the wire-tension data, respectively. The device’s physical size was approximately 15.3 cm × 10.6 cm × 7.5 cm. Two 9 V batteries were used to supply the load cell’s power required. The device prototyped was applied in the field tests of a college-level hammer-throw team. Although there were some limitations, such as the infrared sensor’s problem of acquiring the distance data and the device’s size, the wearable-sensor system has attracted great interests from the coach and athletes for its user-friendly nature in practice. Encouraged by their favorable evaluation and comments, the design of the wearable-sensor system was further improved as shown in Table 2. The microcontroller was replaced from Arduino Mega board to Arduino Pro Mini board, which was aimed for designing our own printed circuit board (PCB) to miniaturize the wearable device. The optical infrared sensor was replaced by a micro IMU to obtain more reliable vertical distance data [8]. An extra IMU was integrated into the system for acquiring the vertical displacements from the wrists to the ground, as such, the full-body kinematics could be revealed. The battery capacity was also expanded to make the device more durable in field training. In addition, an AI module was proposed to estimate the joint angles on the upper and lower limbs by using the wearable-sensor data.

**Figure 1:**
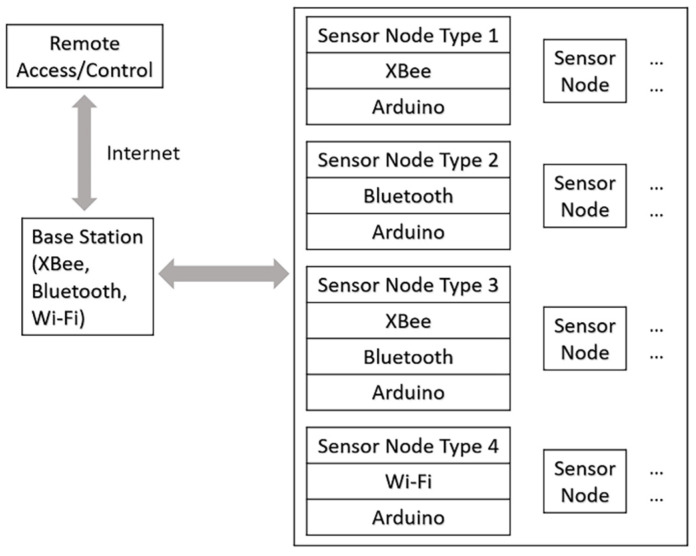
A reconfigurable WSN structure based on Arduino platform (The figure is adapted from the 1st author’s Ph.D. thesis [6]).

In terms of wireless sensor networks (WSNs), four types of the sensor nodes were proposed based on Arduino platform as illustrated in Figure 1. Such kind of reconfigurable WSN structure would allow users to select an appropriate sensor node in different applications. In this study, only the first type of the sensor node was implemented. The XBee data transmission is stable with 50 Hz sampling rate. Its outdoor data transmission range is also much further than Bluetooth and Wi-Fi (i.e., kilometers-level versus 10 m level). These features would be more suitable for field data collection. Digi^®^ XCTU software was used to configure the two XBee modules [31].

**Figure 2:**
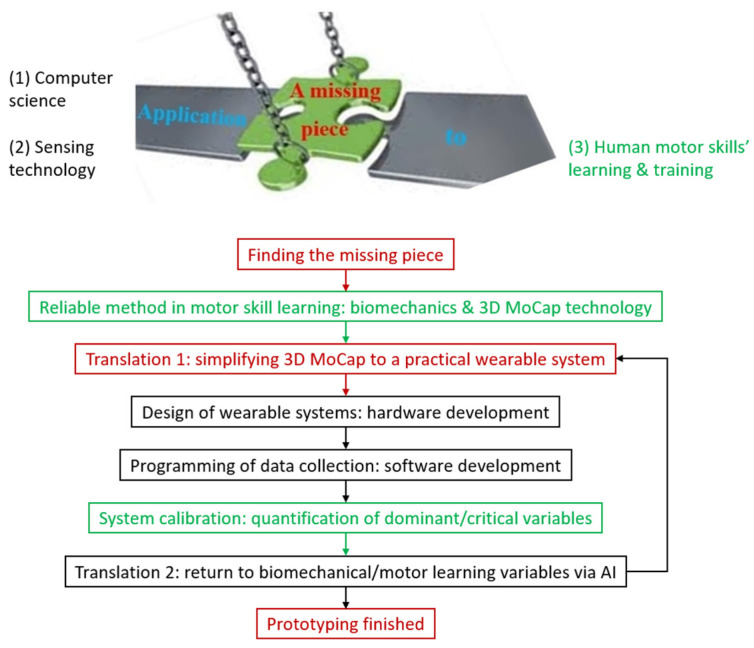
The general modus operandi/framework for developing wearable-sensor systems to help establish real-time biomechanical feedback training in field (The figure is adapted from the 1st author’s Ph.D. thesis [6]).

Finally, the most important aspect of wearable application in sports is its practicality. The recent studies related to elite sports training have found that there is a big gap in developing real-time biomechanical feedback systems [9]. Researchers are still trying to identify the essential biomechanical parameters influencing the elite motor skills, including hammer throw, by using some commercial products, such as Xsens^®^ wearable-sensor system [32]. Therefore, a framework has been devised for developing task-specific wearable-sensor systems to help establish real-time biomechanical feedback training in field, as shown in Figure 2. In essence, the development of biomechanical wearables is to apply the knowledge of computer science and sensing technology (i.e., black font in Figure 2) into human motor learning and training (i.e., green font). Obviously, a connecting piece is missing (i.e., red font) for bridging the gap between the two sides. For well-developed areas, researchers have already established modus operandi to find the missing piece for practitioners/users. Unfortunately, to our best knowledge, such a modus operandi does not exist for the development of biomechanical wearables in elite sports. Therefore, researchers have to find the missing piece first. The method begins with 3D motion capture/biomechanical quantification, followed by finding ways to simplify the 3D motion capture to wearables, design of wearable systems, programming, quantification of dominant variables, and ends up with transitions from the wearables’ data to biomechanical variables (Figure 2). If the transition is successful, the prototyping is finished; otherwise, one should go back for a revision (e.g., adding more sensors) until reaching the accuracy required. Although the framework is derived from the hammer-throw study, the methodology/modus operandi could be extended into various human motor skills.

In summary, this paper presents a further developed wearable technology that leads to high practicality and user-friendly training feedback device. The new development has optimized our previous wearable system and can provide the real-time biomechanical feedback in the hammer throw. The rest contents will be organized as follows: Section 2 will introduce the system’s hardware development and the implementation of the deep learning method. Section 3 will present the results of the deep learning models. Section 4 will be the discussion and draw the conclusion.

## 2. Wearable-Sensor System Design and Development

### 2.1. System Architecture and Hardware Development

**Figure 3:**
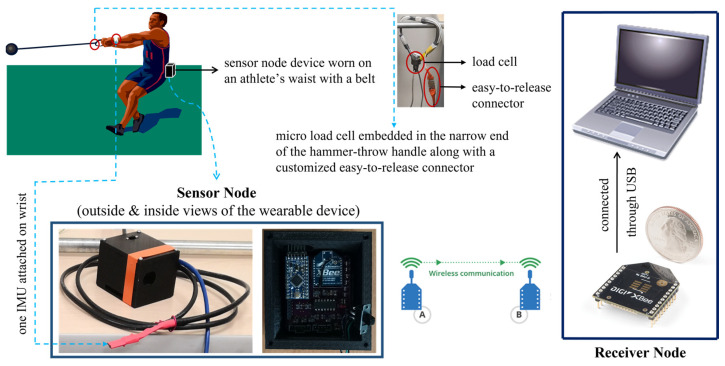
System architecture: the system consists of a sensor node and a receiver node. The sensor node device, including an Arduino microcontroller, an XBee wireless communication module, an on-board IMU (inserted onto the PCB inside the device), an attachable IMU (wrapped with red tape), an embedded micro load cell (along with a customized easy-to-release connector), etc., is worn on an athlete’s waist with a belt, which is used for collecting data in field tests and sending the data to the receiver node. The receiver node has another XBee module connected to an end device (e.g., laptop), which is used for receiving, processing, and analyzing data.

Figure 3 displays the system architecture that has been designed for the real-time biomechanical feedback training in hammer throw. The system consists of a sensor node and a receiver node. The sensor node device can be worn on an athlete’s waist with a belt, which is used for collecting data in field and sending the data to the receiver node. The wearable device’s physical size was miniaturized by designing a customized PCB, where an Arduino microcontroller, an XBee wireless communication module, two IMUs and a micro load cell can be inserted or connected onto the PCB. The optical infrared sensor used in the previous design [31] for acquiring the vertical waist displacements has been replaced by the on-board IMU, because it was found that the infrared sensor could not always be towards ground vertically during a hammer-throw movement, which yielded unreliable datasets. The current design has improved the accuracy of the data with an on-board IMU. Additionally, one more IMU has been added and integrated into the system for collecting the vertical wrist displacements. The micro load cell is still soldered and embedded in the narrow end of the hammer-throw handle. The handle is specially made with an easy-to-release connector, which enables the hammer to be thrown away in a complete hammer-throw movement [31]. The receiver node has another XBee module connected to an end device (e.g., laptop), which can be used to receive, process, and analyze the data obtained by the sensor node device.

**Figure 4:**
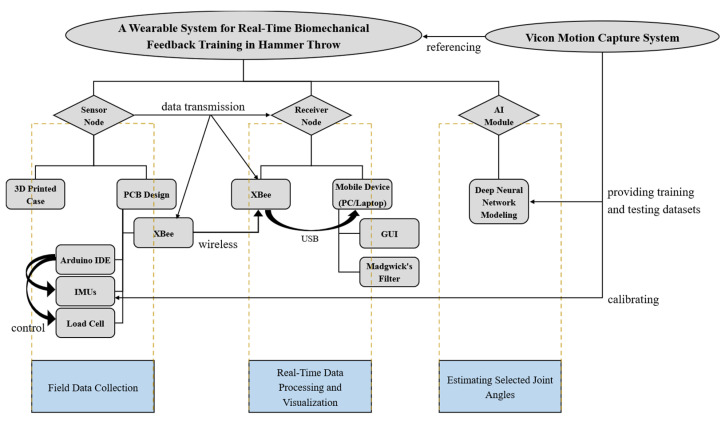
A wearable-sensor system including its hardware and software development is designed for real-time biomechanical feedback training in hammer throw. AI technology is also applied to estimate selected joint angles based on a local motion feature existing among upper and lower limbs during the hammer-throw movements. An optoelectronic motion capture system is used as a referencing system to provide reliable kinematic data.

The flowchart of designing a wearable-sensor system for real-time biomechanical feedback training in hammer throw is illustrated in Figure 4. The sensor node (i.e., the wearable device) consists of a microcontroller (i.e., Arduino Pro Mini 328-5V [33]), a wireless data transmission module (i.e., Digi^®^ XBee 802.15.4 RF module [34]), MEMS (Micro-Electro-Mechanical System) sensors (i.e., two Pololu^®^ MinIMU-9 v5 IMUs [35] and one Omega^®^ LCFD-1k load cell [36]), and other related electronic components. The Arduino microcontroller is tiny, low-cost, and low power consumption, yet it has high performance, which demonstrates the core feature of the Internet of Things (IoT) [37]. A customized PCB and its 3D-printed case have been created to miniaturize the wearable device. The receiver node consists of another XBee module and a mobile device that is used for real-time data processing and data visualization. Madgwick’s filter [38] has been implemented in MATLAB for acquiring the distance data by using IMUs [8]. An AI module (i.e., deep neural network modeling) has been applied in the system for estimating selected key joint angles, which can demonstrate the motion feature existing among upper and lower limbs during the hammer-throw movements. The deep neural networks’ training and testing datasets are provided by Vicon^®^ 3D motion capture system. The Vicon^®^ system is also used as a referencing system to calibrate the IMU device. Due to the limited amount of data, the AI module has not yet been integrated into the system. More details of this issue will be discussed in Discussion.

**Figure 5:**
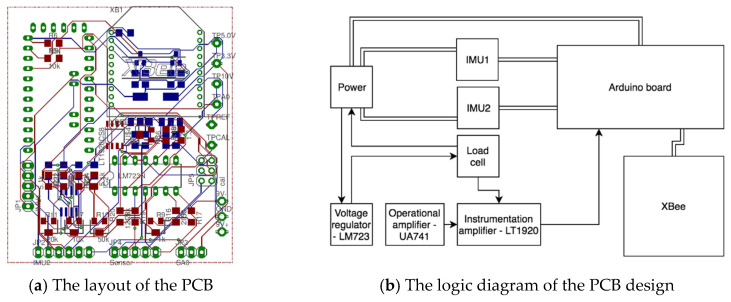
The hardware design of the wearable device: (**a**) The layout of the PCB; (**b**) The logic diagram of the PCB design.

The customized PCB was designed in Altium Designer. Figure 5a displays its layout. It depicts the top copper surface in red, bottom copper in blue, and pads which are visible on either side of the board in green. Besides all the plug-in components on the surface of the PCB, the other electronic components are various resistors, capacitors, potentiometers, three voltage regulators, two MOSFETs (Metal-Oxide-Semiconductor Field-Effect Transistor), an operational amplifier (OPAMP), and an instrumentation amplifier. A calibration module consisting of a voltage divider and a potentiometer allows the load cell to be disconnected from the instrumentation amplifier and be connected to the calibration circuit instead. It provides a range of voltages to the instrumentation amplifier and the Arduino ADC so that the gain and the offset can be adjusted to provide the best signal from the load cell. Figure 5b shows the logic diagram of the major electronic components on the PCB. An 18 V power supply (consisting of twelve 1.5 V AA batteries) is divided into two portions, i.e., providing a +/−9 V dual-rail supply. Two voltage regulators are used to change the power to 5 V and 3.3 V for supplying the Arduino and XBee circuitry, respectively. The IMUs also require 5 V. The additional pin on the second IMU port is required to drive the slave address pin so that the two IMUs could work on the same I2C bus. The voltage regulators are selected so that the required current for all components can be provided by a single 5 V or 3.3 V regulator. The load cell requires a constant and stable 10 V supply because of the power requirements set by the manufacturer. The linear voltage regulator (i.e., LM723 [39]) is used to generate the required 10 V supply. The voltage signal generated from the load cell is too small for the analog to digital converter (ADC) on the Arduino to detect the signal during the calibration procedure. Hence, the instrumentation amplifier (i.e., LT1920 [40]) is required to amplify the incoming signal. The OPAMP (i.e., UA741 [41]), along with the accompanying resistors and potentiometer, produces an offset voltage, allowing the signal to fall within the range of the Arduino’s ADC. In terms of the power consumption of the sensor node, only the load cell’s power consumption needs to be considered, as the other major components are determined to have the feature of low-power consumption. The load cell requires a 10 V excitation, and its minimum bridge resistance is about 10,000 ohms. With the help of an online force sensor power consumption calculator, its power consumption is 10 mW. Therefore, the sensor node device will be able to keep working for weeks with AA batteries (i.e., about 2000 to 3000 mAh).

A 3D-printed case has been designed in Autodesk Inventor to be able to place the PCB, the sensors, and other components inside the case. The device’s outer dimensions are 8.6 cm × 7.8 cm × 5.3 cm, which is nearly half size of the previous design. A space is reserved on the lid of the device for installing a switch. Another space is also reserved under the PCB at the bottom of the device for putting a belt through the case to make it wearable conveniently.

Each sensor has been tested for accuracy before being installed into the device. Because the IMUs have already been calibrated and tested with the updated microcontroller [8], no repeated calibration is needed in this paper. The accuracy of the IMUs has been determined to be 6% after calibration which is done with several synchronized tests with Vicon^®^ 3D motion capture system. The calibrated IMUs are accurate enough in sports skill related motion analysis. Regarding the load cell, thanks to its high linearity, a linear regression method is used to obtain its calibration equation. During the calibration experiments, the hammer-throw handle was hanging freely. A cable was tied on the handle to hang different dumbbell plates as a simulation of different wire-tension values. The initial weight was zero. The weights were added by 5 lb every time until 35 lb. For every 5 lb, the raw data were recorded. The unit of the weight was converted from lb to kilogram (kg) as a convention (i.e., 0 kg, 2.27 kg, 4.54 kg, 6.8 kg, 9.07 kg, 11.34 kg, 13.61 kg, 15.88 kg). Eventually, the slope and intercept of the linear regression equation were calculated [42].

### 2.2. Deep Learning Model

**Figure 6:**
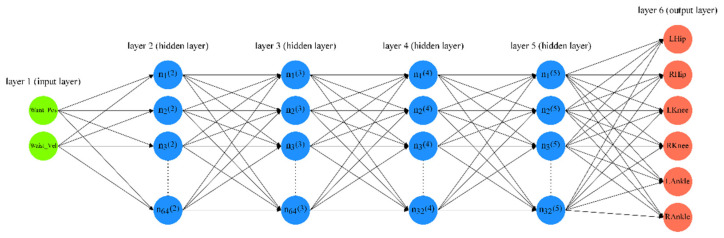
The deep neural network structure of the simplified model.

Based on the Keras API imported from the TensorFlow open-source framework [43], two Sequential Regression models have been implemented in Jupyter Notebook to verify if the vertical wrist and waist/hip displacements can be used to reveal the upper and lower limb motor coordination. One model was defined as a simplified model estimating selected joint angles on lower limbs only, whereas the other one was defined as a complete model estimating selected joint angles on both upper and lower limbs. The deep neural network (DNN) structure of the simplified model is shown in Figure 6. The structure of the complete model is just similar, so it is not illustrated here.

Four densely connected hidden layers (i.e., dense layers) have been set. In each dense layer, a rectified linear unit (ReLU) activation function has been used for both models [44]. The function’s output will be passed into the next layer as an input, or it will be the final output. In this way, ReLU has the advantage of increasing the sparsity of a neural network because some neuron’s outputs can be zero. In other words, it helps to reduce the dependence of each parameter to avoid overfitting [45]. The optimizer has been set to Adam, which supplies the best effectiveness in this case in comparison to other optimizers such as RMSProp [46,47]. Additionally, by comparing different values, the learning rate is set to 0.0007 for both models. Mean squared error (MSE) has been used as the loss function. Mean absolute error (MAE) and MSE have been applied as the metrics.

The datasets were collected from a college-level athlete by using Vicon^®^ 3D motion capture system. The research protocol was scrutinized by the Human Subjects Research Committee of the University of Lethbridge/Canada. The committee has confirmed that the experimental protocols meet the criteria of ethical conduct for research involving humans. The athlete was informed that the device would be used for collecting data related to their throws. The subject signed the approved consent form and voluntarily participated in the data collection. A full-body 3D kinematic data (including all joints/segmental angles) were quantified by using 39 tracking markers (9 mm diameter). Because an athlete always holds the hammer’s handle with two hands together until releasing the hammer, the data of either side of the wrists can be used as an input variable. The 3D data have shown that the difference between two wrists’ data is so trivial that it can be neglected in the hammer-throw analysis. In our test, the data of the left wrist have been selected. Thus, the average of the trajectories of the left wrist thumb side (i.e., LWRA) and the left wrist pinkie side (i.e., LWRB) on Z axis (vertical to ground) was used to denote the vertical wrist displacements. Similarly, the average of the trajectories of the left anterior superior iliac spin (i.e., LASI) and the right anterior superior iliac spin (i.e., RASI) was used to denote the vertical hip/waist displacements. In addition to the position values, the velocity values were also calculated for increasing the data volume. As the sampling rate was set to 200 Hz, the velocity values were calculated as in:

vel_i_ = (pos_i+1_ − pos_i_) ÷ 0.005, (1)

where vel_i_ was the velocity at time i, pos_i_ was the position at time i, and pos_i+1_ was the position at the next timestamp. In total, four groups of input variables were used in the two models, including the wrist displacements/positions (i.e., Wrist_Pos), the wrist velocities (i.e., Wrist_Vel), the hip displacements/positions (i.e., Waist_Pos), and the hip velocities (i.e., Waist_Vel). The simplified model’s input variables were Waist_Pos and Waist_Vel, and its output variables were the left hip angle (LHA), the right hip angle (RHA), the left knee angle (LKA), the right knee angle (RKA), the left ankle angle (LAA), the right ankle angle (RAA). The complete model had seven more output variables including the left shoulder angles (LSA), the right shoulder angles (RSA), the left elbow angles (LEA), the right elbow angles (REA), the left wrist angles (LWA), the right wrist angles (RWA), and the thorax angles (TA). The data were randomly separated into two groups with a factor of 0.8 to form the training datasets and the testing datasets.

## 3. Results

### 3.1. Calibration for the Load Cell

By calibrating and testing the load cell, a calibration equation has been established as follows:

W = 0.51225 × R − 3.03672,(2)

where R referred to the raw data and W referred to the actual weight values with unit in kg. Afterwards, the calibration equation has been doubly checked by using 45 lb (20.41 kg) and 55 lb (24.95 kg). The absolute errors are 0.12 kg and −0.07 kg, respectively, returning a mean relative error of 0.87%. This result demonstrates the load cell’s high linearity. Therefore, this equation has been applied in the system software to convert raw data (i.e., voltage) to conventional tension/force data.

### 3.2. Deep Learning

**Figure 7:**
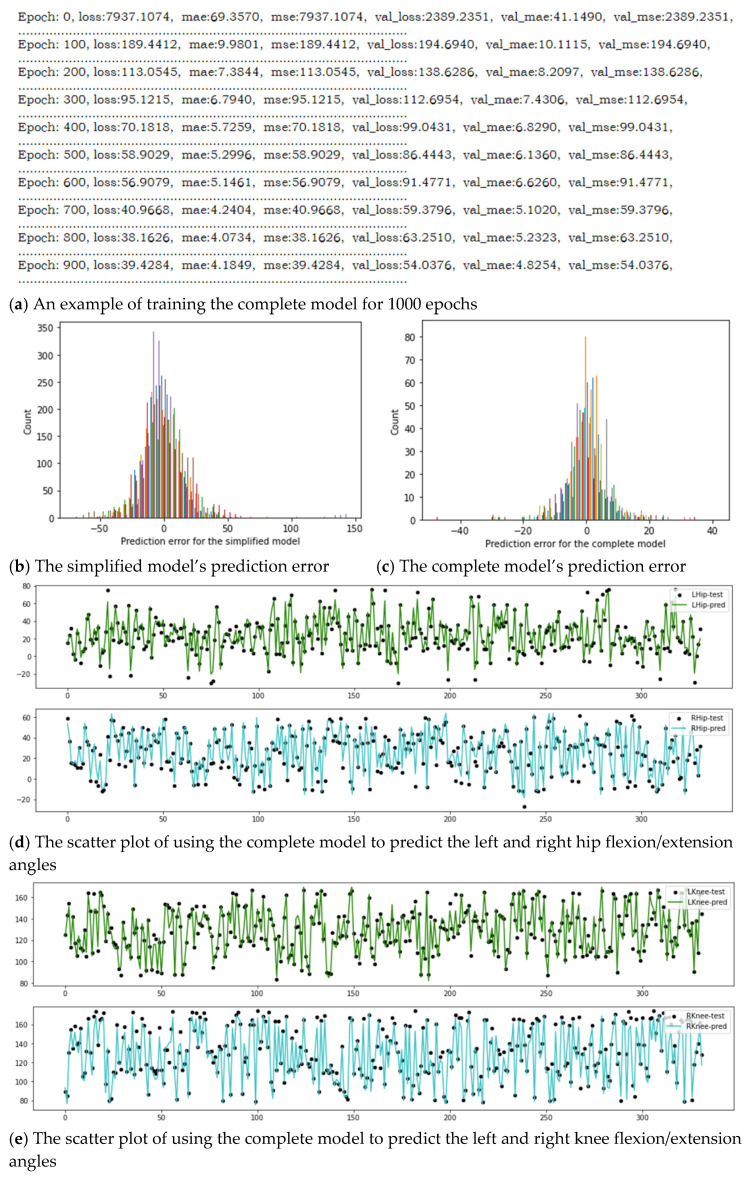

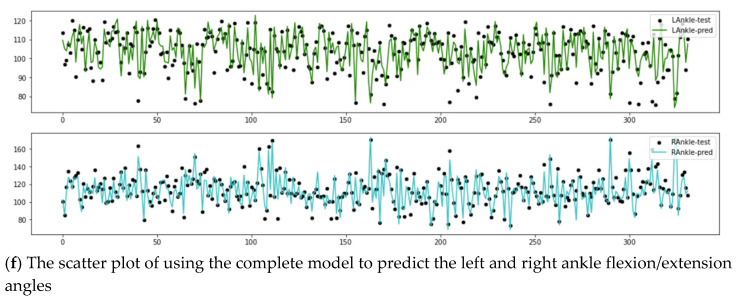
A typical result of the DNN modeling: (**a**) An example of training the complete model for 1000 epochs; (**b**) The simplified model’s prediction error; (**c**) The complete model’s prediction error; (**d**) The scatter plot of using the complete model to predict the left and right hip flexion/extension angles; (**e**) The scatter plot of using the complete model to predict the left and right knee flexion/extension angles; (**f**) The scatter plot of using the complete model to predict the left and right ankle flexion/extension angles.

Both models have been trained for 1000 epochs to reach convergence. The simplified model’s loss stopped decreasing at around 250 whereas the complete model’s loss stopped decreasing at around 30. Figure 7a just shows an example of training the complete model. The training procedure of the simplified model is just similar. After training the two models, they have been evaluated with the testing datasets. As illustrated in Figure 7b,c, the prediction errors for both models satisfy the Gaussian distribution. Figure 7d–f demonstrate the scatter plots of using the complete model to predict the flexion/extension angles on hip, knee, and ankle, respectively. Again, the simplified model’s prediction results are just similar. So, they are not shown here. The results of the DNN models have further indicated that the upper and lower limb coordination can be revealed by the strong correlation existed between the selected vertical displacements and the selected joint angles [8,48].

The MAEs and MSEs of the two models are displayed in Table 3. The MAEs for both models are much better than the MSEs due to the outliers in the training datasets. In Table 3, Model I is the simplified model using for estimating LHA, RHA, LKA, RKA, LAA, and RAA. Model II is the complete model using for estimating LHA, RHA, LKA, RKA, LAA, RAA, LSA, RSA, LEA, REA, LWA, RWA, and TA. In order to compare the two models more meaningfully and fairly, only the results from the lower limbs (i.e., Hip, Knee, Ankle) of the complete model have been selected for comparisons. The comparisons have shown that the simplified model has estimated the hip flexion/extension angle of approximately 12 degrees, the knee flexion/extension angle of approximately 14 degrees, and the ankle flexion/extension angle of approximately 10 degrees; while the complete model has estimated the hip flexion/extension angle of approximately 4 degrees, the knee flexion/extension angle of approximately 4 degrees, and the ankle flexion/extension angle of approximately 5 degrees. Obviously, the complete model has outperformed the simplified model. It might be caused by the data diversity; i.e., the complete model has two more inputs and seven more outputs than the simplified model. To justify this result, normalization of the datasets has been tried but returned poor results. The average accuracy (i.e., MAE) of the simplified model has been improved by 67.4% and the average accuracy (i.e., MAE) of the complete model has been improved by 78.8%, when comparing to using unnormalized data. So, the normalization of the datasets might reduce the diversity of the data to some extent.

## 4. Discussion

When performing complex sport skills, such as hammer throw, both upper and lower limbs must coordinate; the coordination is simply too fast and complicated to be analyzed without motion capture technology, including wearable-based technology. It is commonly acknowledged that wearable technology represents the future in this area. On the other hand, as stated in the Introduction, the two vital aspects for developing wearable technology in complex sport skills’ training are practicality and user-friendly of a product. As a pilot study on the highly complicated hammer throw, the current study and its prototype have successfully established a framework which future developments of wearable technology in various elite sports could adapt and/or make adjustments to. The following are some unique features that can be meaningful to sports applications.

### 4.1. Issues Related to Practicality of Wearable Technology

It is ideal that only one wearable sensor unit can supply biomechanical information related to motor control, just like one physiological wearable device can show heart rate and blood pressure regardless the type of movement. Unfortunately, the information related to limbs’ coordination (i.e., motor control) often differ from one skill to the other. This means that biomechanical wearable device must be tailored to an individual sport being examined. It is nearly impossible to use one wearable sensor to quantify motor control of a complex sports skill. If people want to obtain more kinematic and kinetic data, more wearable sensors will be required. Nevertheless, due to the hardware limitations (i.e., the sensors’ size, weight, rigidity, etc.), too many wearable sensors will induce movement constraints, resulting non-reliable analysis. Consequently, the wearable system developed will be useless/impractical for practitioners. The results of the current study have highlighted one solution for reaching high practicality in the development of wearable systems, i.e., combining wearables with AI technology. Since the tension sensor is implanted in the handle and wearing the two IMUs has neglectable effect on the movement control of the hammer throw, the practicality of the device is indeed high. Our future effort will focus on minimizing the data collecting unit to improve the practicality further.

Soft and flexible IMU sensors might be another solution [49,50,51]. Placing some soft and flexible IMU sensors on body during the fast and complex motor skills may not affect athletes’ performance too much. However, the concern is how to improve the accuracy and reliability of the soft and flexible PCB and sensors. Future studies may explore this possibility.

### 4.2. Potential of AI in the Development of Biomechanical Wearables

**Figure 8:**
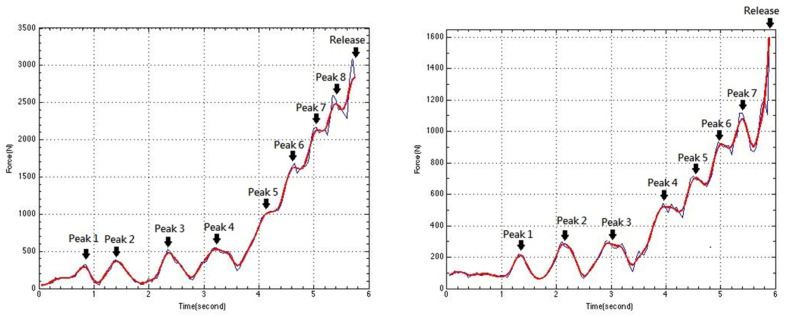
The two patterns of tension development during throwing: gradually increasing during body rotation (**left** in the figure, Peak 5–8) and suddenly increasing toward the end (**right** in the figure) (the figure is adapted from the 1st author’s PhD thesis [6]).

In addition to reduce the number of sensors, AI has the other advantage in the development of biomechanical wearables: synchronizing the kinematic and kinetic data analysis. This is especially relevant for a user-friendly product. Individualized optimization is always performed by coaches because various control patterns are developed based on individual anthropometry, power, reaction and more. Our tension data have identified two different tension-development patterns (Figure 8). It is well known that the tension generation (kinetic data) is influenced by limbs’ coordination (kinematic data). It is vital for coaches to analyze the two types of data together for making control optimization. It is the first time that the two data synchronously obtained by wearable device, thanks to the AI technology.

### 4.3. Limitations and Suggestions

This is the first study that explore the wearables with AI support in application of complicated sports skills analysis. It is understandable that there are limitations associated with this study. The obvious one is the data volume for deep learning models’ training. The simplified model has more than twice as much data as the complete model did. However, the data volumes of the two models are still in the same level (i.e., between 10 thousand and 100 thousand). Since it is quite difficult to set up optoelectronic motion capture system in field to obtain “big data”, the DNN models might not fully reveal their advantages. Therefore, even though the simplified model has more training datasets, its results might not be as decent as the complete model’s results. By further examining the data, much more outliers exist in the simplified model than in the complete model. If an algorithm can be designed and implemented to remove the outliers, the performance of the simplified model might be improved. Considering the practicality and reliability of the method proposed in this study, building more simplified models would still be recommended instead of building a model with more sensors. As discussed above, the reason is that to realize real-time biomechanical feedback training, fewer sensors should be applied to reduce the movement constraints to athletes. For a compromised exploration, one could try to integrate another IMU into the system. By putting it on the back of an athlete, another simplified model (i.e., upper-body model) can be built. Thus, the new simplified model can be compared with the complete model again as a further validation. In addition, only one athlete’s data are processed and analyzed with Vicon Nexus software due to the time-consuming issue. To make full use of the DNNs, more athletes’ data should be required to be processed for increasing the data volume.

Following our pilot study on a multimodal wearable system by applying a dual-chain biomechanical model [52], a vertical GRF measurement module will be integrated into the system for the next step. Moreover, it is also one of our research goals to use surface electromyography to extract different motion features from different motor skills so that some important motion patterns can be figured out to reveal the inter-/intra-limb motor coordination.

## 5. Conclusions

Currently, it is still difficult to obtain reliable kinematic and kinetic data in field/real competition environment, leading to the lack of real-time biomechanical feedback training studies in sports areas. This paper has pioneered a wearable-sensor system combining with AI technology for providing real-time biomechanical feedback in the hammer-throw training. The pilot study is the first practical research in this field to our best knowledge. Although it is still a prototype and needs further development, the idea of combining wearables with AI has been proved to have great potential in the hammer-throw field tests and can be extended to daily trainings of different sports. The framework for developing such wearable biomechanical feedback systems in any elite sports has been built. Therefore, in the future, more studies could be conducted by following the framework for developing practical and user-friendly systems in different sports. As more and more developments of biomechanical wearables in the future, human motor skill learning and training will be transitioned from experience-based endeavor to evidence-based practice.

## Figures and Tables

**Table 1:** The comparison of three types of biomechanical feedback approaches/systems.

Traditional Optoelectronic Systems	Computer-Vision Based Markerless Systems	Wearable Systems
Movement constraints caused by at least ~40 markers for a full-body motion capture	No movement constraints to athletes	Movement constraints caused by at least 17 wearable sensors for a full-body motion capture
Accuracy of the markers’ trajectories can be within 1 mm	Accuracy of the virtual markers can be in millimeter level	Accuracy of the sensors’ trajectories can be in centimeter level
Usually lab-based capturing	Usually within 5 m * 5 m’ capturing space but can be expanded with AI modeling	No limitations for the capturing space
Easy but complicated calibration work	Difficult and complicated calibration work	Easy and convenient calibration work
Time-consuming course on data processing	Real-time monitoring but requiring high-quality hardware and high-level AI modeling	Real-time monitoring

**Table 2:** The comparison of the features between the previous design and the current design of the wearable-sensor system.

	Features	Previous Study	Current Study
Hardware	Microcontroller	Arduino Mega	Arduino Pro Mini
	Wireless communication	XBee RF modules	XBee RF modules
	Sensors	Load cell & optical infrared sensor	Load cell & IMUs
	PCB design & 3D printing	No	Yes
	Physical size	15.3 cm × 10.6 cm × 7.5 cm	8.6 cm × 7.8 cm × 5.3 cm
	Battery capacity	9 V batteries—about 500 mAh	AA batteries—about 2000 to 3000 mAh
Software	Algorithm	N/A	Madgwick’s filter for IMU data fusion
	AI module	N/A	Deep neural network models built offline

**Table 3:** The results of comparing the simplified model with the complete model estimating the selected joint angles on lower limbs.

		Hip		Knee		Ankle	
		Left	Right	Left	Right	Left	Right
**Model I**	**MAE**	~11°	~12°	~13°	~14°	~9°	~11°
	**MSE**	~241°	~255°	~491°	~557°	~310°	~439°
**Model II**	**MAE**	~4°	~4°	~4°	~4°	~5°	~5°
	**MSE**	~37°	~26°	~26°	~39°	~41°	~57°

## Data Availability

The data supporting the findings of this study are available on reasonable request from the corresponding authors. It is not publicly available due to privacy or ethical restrictions.
